# Caging and photo-triggered uncaging of singlet oxygen by excited state engineering of electron donor–acceptor-linked molecular sensors

**DOI:** 10.1038/s41598-022-15054-4

**Published:** 2022-07-05

**Authors:** Devika Sasikumar, Yuta Takano, Hanjun Zhao, Reiko Kohara, Morihiko Hamada, Yasuhiro Kobori, Vasudevanpillai Biju

**Affiliations:** 1 Graduate School of Environmental Science, Hokkaido University, N10, W5, Sapporo, 060-0810 Japan; 2 Research Institute for Electronic Science, Hokkaido University, N20, W10, Sapporo, 001-0020 Japan; 3 Department of Chemistry, Graduate School of Science, Kobe University, 1-1 Rokkodaicho, Nada-Ku, Kobe, 657-8501 Japan

**Keywords:** Chemistry, Organic chemistry, Photochemistry, Physical chemistry

## Abstract

Singlet oxygen (^1^O_2_), one of the most sought-after species in oxidative chemical reactions and photodynamic cancer therapy, is activated and neutralized in the atmosphere and living cells. It is essential to see "when" and "where" ^1^O_2_ is produced and delivered to understand and utilize it. There is an increasing demand for molecular sensor tools to capture, store, and supply ^1^O_2_, controlled by light and engineered singlet and triplet states, indicating the ^1^O_2_-capturing-releasing state. Here, we demonstrate the outstanding potential of an aminocoumarin-methylanthracene-based electron donor–acceptor molecule (**1**). Spectroscopic measurements confirm the formation of an endoperoxide (**1-O**_**2**_) which is not strongly fluorescent and remarkably different from previously reported ^1^O_2_ sensor molecules. Moreover, the photoexcitation on the dye in **1-O**_**2**_ triggers fluorescence enhancement by the oxidative rearrangement and a competing ^1^O_2_ release. The unique ability of **1** will pave the way for the spatially and temporally controlled utilization of ^1^O_2_ in various areas such as chemical reactions and phototherapies.

## Introduction

Singlet oxygen (^1^Δ_*g*_) (^1^O_2_), the lowest excited state of molecular oxygen, is an essential member of the reactive oxygen species (ROS) family and an active intermediate in various chemical and biological reactions^1–5^. Uncontrolled production of ^1^O_2_ causes undesirable degradation of materials and oxidative stress-induced disease progression. Therefore, the controlled and localized generation and sensing of ^1^O_2_ are essential and beneficial for utilizing ^1^O_2_ in chemical and biological reactions arbitrary.

^1^O_2_ sensing is important to detect and control its reactions, such as in PDT to kill cancer cells or in fine chemicals synthesis^1–4,6^. Fluorogenic sensing is one of the most efficient methods for ^1^O_2_ detection because of its high sensitivity^5,7^. One of the most promising fluorescence sensors of ^1^O_2_ is based on a fluorophore-spacer-^1^O_2_ receptor system. Anthracene moiety is often chosen as an excellent receptor owing to its high selectivity and reactivity toward ^1^O_2_^8^. Such sensors are nonfluorescent before reacting with ^1^O_2_ due to efficient photoinduced intramolecular electron transfer (PET). Cycloaddition between ^1^O_2_ and a sensor affords an endoperoxide, blocking the PET and uncaging the emission^5^.

Apart from the ^1^O_2_ sensing, the demand for capturing and controlled releasing of ^1^O_2_ has also been studied significantly in various areas in biology^1–3^ and chemistry^4^. However, it is challenging to produce it in the hypoxic tumor microenvironment^1–3^. This challenge is examined by stimuli-induced releasing of ^1^O_2_^9–16^. Conventionally, ^1^O_2_ was liberated by heating endoperoxides of acenes or piperidones^10–14^. Fudickar et al. developed a dipyridylanthracene endoperoxide and released ^1^O_2_ under a chemical trigger^13^. Ucar et al. demonstrated a two-step chemical stimulus-induced release of ^1^O_2_ from naphthalene endoperoxide^14^. Phototrrigered ^1^O_2_ release is also reported by the photoexcitation on the anthracenyl part of the endoperoxide, although the high energy light of 282 nm laser was used^16^. Despite many ^1^O_2_ sensor molecules reported^17–21^, the sensor shows unexpected capture, store, and supply ^1^O^2^, indicating the capturing-releasing states with > 50-fold fluorescence intensity enhancement from the D-A form to the caged form and the endoperoxide.

Herein, the present study demonstrates the molecular dyad system that chemically traps, optically releases, and efficiently senses ^1^O_2_ in a temporally controlled manner. It is identified that an aminocoumarin-methyl anthracene-linked molecule (**1**) traps ^1^O_2_ to form the endoperoxide (**1-O**_**2**_). Remarkably, **1-O**_**2**_ is not as fluorescent as commercially available fluorogenic ^1^O_2_ probe molecules with an anthracenyl moiety. It is indicated in the present study that the unique molecular orbitals and triplet excitation energy levels of **1-O**_**2**_ offer a weak fluorescent nature. An additional UV or NIR light-stimuli triggers the formation of a highly fluorescent compound. We also confirmed that **1-O**_**2**_ releases ^1^O_2_ by photoexcitation on the dye molecule, coumarin, by one- or two-photon (near-infrared, NIR) excitation. The unique excited states originate from the aminomethyl anthracenyl moiety in 1-O_2_, making the efficient ^1^O_2_ capturing, storing, and releasing with fluorescence sensing achievable by one- or two-photon excitation. These unique phenomena are verified using spectroscopic measurements, including NMR and EPR, and density functional theory (DFT) calculations.

## Methods

### General

All chemicals used in this research were analytical grade and used as received unless otherwise stated. Potassium carbonate (K_2_CO_3_), potassium iodide (KI), hydrochloric acid (HCl), and sodium azide (NaN_3_) were obtained from FUJIFILM Wako Pure Chemical Corporation, Japan. 7-Amino-4-methyl coumarin, 7-ethylamino-4-methyl coumarin, 9-chloromethyl anthracene, 9-methylanthracene, tetrakis(4-carboxyphenyl)porphyrin (TCPP), and Rose Bengal (RB) from Tokyo Chemical Industry (TCI), Japan. We obtained 2,2,6,6-tetramethylpiperidine (TEMP) and 2,2,6,6-tetramethylpiperidine-1-oxyl (TEMPO) from Sigma Aldrich, USA. SOSG was obtained from Sigma and SiDMA sensor from DOJINDO, Japan. All the solvents were in reagent grade and from FUJIFILM Wako Pure Chemical Corporation, Japan.

Absorption spectra were recorded using an Evolution 220 UV–visible spectrophotometer (ThermoFisher Scientific), and fluorescence (FL) spectra were recorded using a Hitachi F-4500 FL spectrofluorometer. NMR measurements were performed using an Agilent Unity INOVA 500 or JEOL ECX-400 spectrometers. The continuous-wave-EPR measurements were carried out using a Bruker EMXplus spectrometer. For photoirradiation of samples, we used a DPSS 532 nm Green laser (Shanghai Dream Laser Technology), a Xenon/ Mercury lamp (Hamamatsu Photonics KK, Japan), or an 800 nm femtosecond laser (Coherent Mira 900, the pulse width is 140 fs). The 404 nm laser (Thorlabs, 600 mW) with neutral density filters were used for varying the power.

### Synthesis and characterization

**1** was prepared and characterized according to the reported procedure with slight modification^20^. 7-amino-4-methylcoumarin (0.175 g, 1.00 mmol) and 9-chloromethylanthracene (0.227 g, 1.00 mmol) were dissolved in 20 mL of acetonitrile. Then, DBU (304 mg, 2.00 mmol) was added to the solution, and the reaction mixture was stirred at 82 °C for 6 h. The reaction mixture was cooled to room temperature, and excess water was added, which provided a yellow residue. The pH of the solution was adjusted to ~ 6–7 using aq. HCl. The residue was filtered and dried. The yellow powder was re-dissolved in hot THF and then reprecipitated by adding an excess of toluene, and the residue was filtered and washed with toluene and then with acetone to give a pale-yellow powder (0.332 g, 92%). *λ*_max_ (DMF): 354, 370, 389 nm. **1:** ^1^H NMR (400 MHz, CDCl_3_) *δ* = 8.50 (1H), 8.21 (2H), 8.05 (2 H), 7.57–7.40 (m, 5H), 6.74 (1H), 6.58 (1H), 6.02 (s, 1H), 5.21 (2H), 4.30 (1H), 2.40 (3H).

**2** was prepared according to the process as follows.

7-(Ethylamino)-4-methylcoumarin (0.10 g, 0.49 mmol) and 9-chloromethylanthracene (0.16 g, 0.73 mmol) were dissolved in 10 mL of DMF. Then, K_2_CO_3_ (47 mg, 2.9 mmol) and potassium iodide (5 mg, 0.03 mmol) were added to the solution, and the reaction mixture was stirred at 85 °C for 5 h. The reaction mixture was cooled to room temperature, and excess water was added, which provided a yellow residue. The pH of the solution was adjusted to ~ 6–7 using aq. HCl. The residue was filtered and dried. The yellow powder was re-dissolved in hot THF and then reprecipitated by adding an excess of toluene, and the residue was filtered and washed with toluene and then with acetone to give a pale-yellow powder (0.10 g, 51%). *λ*_max_ (DMF): 354, 370, 389 nm. ^1^H NMR (500 MHz, CDCl_3_) δ = 8.52 (s, 1H; Ar–H), 8.14–8.16 (d, 2H; Ar–H), 8.05–8.07 (d, 2H; Ar–H), 7.55–7.48 (m, 5H; Ar–H), 6.95–6.98 (dd, 1H; Ar–H), 6.91–6.92 (d, 1H; Ar–H), 6.05 (s, 1H; allylic), 5.37 (s, 2H; N-CH_2_), 3.06–3.10 (q, 2H; N-CH_2_), 2.42 (s, 3H; CH_3_), 0.77–0.80 (t, 3H; CH_3_).

### Steady-state FL and absorption spectroscopic studies of ^1^O_2_ sensing

A sample solution of a sensor molecule (**1** or **2**; 10.0 μM) and a photosensitizer (5.00 μM) in DMF was photosensitized under selective photosensitizer excitation. The sample solution containing RB was irradiated with a 532 nm (DPSS, 50 mW) continuous-wave laser. That containing TCPP was illuminated with a xenon lamp fitted with a 410–430 nm bandpass filter or a 404 nm (Thorlabs, 70 mW) continuous wave laser. The sample solution was irradiated with a UV LED lamp (Asahi-spectra. Co. CL) (365 nm, 10 nm band path, 1.0 mW cm^-2^). The FL and absorption spectra were recorded before and after irradiation.

### Isolation and characterization of the product of the reaction of 1 and ^1^O_2_

2.0 mM of **1** and 1.0 mM of RB were mixed in 800 μL of DMF (HPLC grade) and illuminated by a green diode laser (532 nm, 50 mW, 10 min). The reaction mixture was subjected to an HPLC system (Agilent 1220) equipped with C18-MS-II column (Nacalai; 4.6 mm I.D. × 250 mm) using DMF as the eluent. The fraction with the peak retention time of 2.8 min was collected and removed the solvent in a vacuum in the dark. DMSO-d_6_ was added and measured by an NMR spectrometer. Yield 86% (estimated from the HPLC profile). *λ*_max_ (DMF): 354 nm, ^1^H NMR (400 MHz, DMSO-*d*_6_) *δ* = 7.53–7.56 (m, 4 H), 7.49 (s, 1H), 7.31–7.33 (m, 4H), 6.98 (d, *J* = 9.1 Hz, 1H), 6.92 (s, 1H), 6.86 (t, *J* = 4.1 Hz, 1H), 6.47 (s, 1H), 5.98 (s, 1H), 4.50 (d, *J* = 4.1 Hz, 2H), 2.35 (s, 3H).

The reaction mixture without the HPLC separation were also measured for comparison. And then, the sample solutions were irradiated with a UV LED lamp (Asahi-spectra. Co. CL) (365 nm, 10 nm band path, 1.0 mW cm^-2^, 10 min) and again measured by the NMR spectrometer. The results were shown in Figs S6 and S7.

### Estimation of the singlet oxygen photo-releasing quantum yield from the EPR)studies

The singlet oxygen photo-releasing quantum yield is estimated to be 1.6% based on the absorbed number of photons (320 nmol) and the detected ^1^O_2_ (10.0 nmol × 50% = 5.00 nmol). The absorbed number of photons was calculated based on the induced light and absorbancy of the sample solution after the photosensitization of RB. The detected ^1^O_2_ in mole was calculated based on the used **1** (10 μM, 0.50 mL) and the signal change ratio of TEMP to be TEMPO (50%).

### Steady-state FL and absorption spectroscopic studies for ^1^O_2_ releasing

A sample solution of **1** (10.0 μM) and a photosensitizer (5.00 μM) in DMF was photosensitized under selective photosensitizer excitation. The sample solution containing RB was irradiated with a 532 nm (50 mW) continuous-wave laser. Then, SOSG (10 μM) was added and the sample solution was irradiated with a UV LED lamp (Asahi-spectra. Co. CL) (365 nm, 10 nm band path, 1 mW cm^-2^). Before adding SOSG, the solution was purged with argon (50 mL/min, 20 min) for the condition of the absence of oxygen. The FL and absorption spectra were recorded before and after irradiation.

### Steady-state FL and absorption spectroscopic studies under NIR-activation of the intermediate complex

A sample solution containing **1** (10.0 μM) and rose bengal (10.0 μM) in DMF was irradiated under a 532 nm green laser (50 mW) for the photosensitized generation of ^1^O_2_. The sample's FL and absorption spectra (250 μL in 5 mm pathlength cuvette) were recorded before and after 30 min of the photosensitization. Then, the sample solution was irradiated with an 800 nm fs laser (Coherent Mira 900) for 40 min, and FL and absorption spectra were recorded every 5 min interval. The samples' FL quantum yield was estimated by a relative FL quantum yield estimation using coumarin 120 as a reference. A control experiment was conducted to compare the enhancement factor by recording the FL spectra of the equivalent sample solution after photosensitization, which was kept under dark.

### Steady-state FL and absorption spectroscopic studies of ^1^O_2_ sensing

A sample solution of a sensor molecule (**1** or **2**; 10.0 μM) and a photosensitizer (5.00 μM) in DMF was photosensitized under selective photosensitizer excitation. The sample solution containing RB was irradiated with a 532 nm (DPSS, 50 mW) continuous-wave laser. That containing TCPP was illuminated with a xenon lamp fitted with a 410–430 nm bandpass filter or a 404 nm (Thorlabs, 70 mW) continuous wave laser. The sample solution was irradiated with a UV LED lamp (Asahi-spectra. Co. CL) (365 nm, 10 nm band path, 1.0 mW cm^-2^). The FL and absorption spectra were recorded before and after irradiation.

### Density functional theory (DFT) calculations

The molecular structures and electron energies were optimized and obtained by the Gaussian16 package^22^ using ub3lyp/6–311 +  + G** level of theory^23,24^. Molecular orbital analyses were performed for the natural transition orbitals^25^ using “Pop = (NTO,SaveNTO)” and “Density = (Check,Transition = *n*)” keywords after performing TD-DFT calculations.

### Electron paramagnetic resonance (EPR) studies

The generation of ^1^O_2_ was indirectly monitored by using a spin probe TEMP that undergoes oxidation by ^1^O_2_ to form EPR-active TEMPO. The measurement conditions were optimized by evaluating the photosensitization of RB in the presence of TEMP. To this purpose, 5 mM of TEMP was added to 5.00 μM RB in DMF. The solution was irradiated with a xenon lamp fitted with > 480 nm long-pass filter for 30 min (50 mW at 532 nm). After the photosensitization, the EPR spectra of the sample solution were recorded using the X band frequency of microwave (9.79 GHz) at 1 mW cm^−2^ power. To check the possibility of generation ^1^O_2_ under UV illumination, **1** or RB was illuminated with a UV lamp with an emission maximum at 365 nm, at 2.0 mW cm^−2^ for 10 min in the presence of 5.00 mM of TEMP.

To examine the UV-activated release of ^1^O_2_, a sample solution containing **1** (10 μM) and RB (5 μM) was irradiated with a xenon lamp fitted with > 480 nm long-pass filter for 30 min (50 mW at 532 nm). After the photosensitization and generation of the intermediate complex, 5 mM of TEMP was added to the sample solution, and EPR spectra were recorded before and after 10 min of UV illumination (365 nm, 10 nm band path, 2 mW cm^−2^) . A control experiment was conducted by illuminating a sample solution containing **1** (10 μM) and RB (5 μM) and 5 mM of TEMP with UV light (UV, 2 mW cm^−2^ at 365 nm).

The enhancement factor of the EPR signals was determined by assuming the formation of TEMPO in the presence of TEMP and RB without **1** to be 100% (Fig. S7).

## Results and discussion

### Reactions of compound 1 with ^1^O_***2***_ generated by a photosensitizer

**Figure 1:**
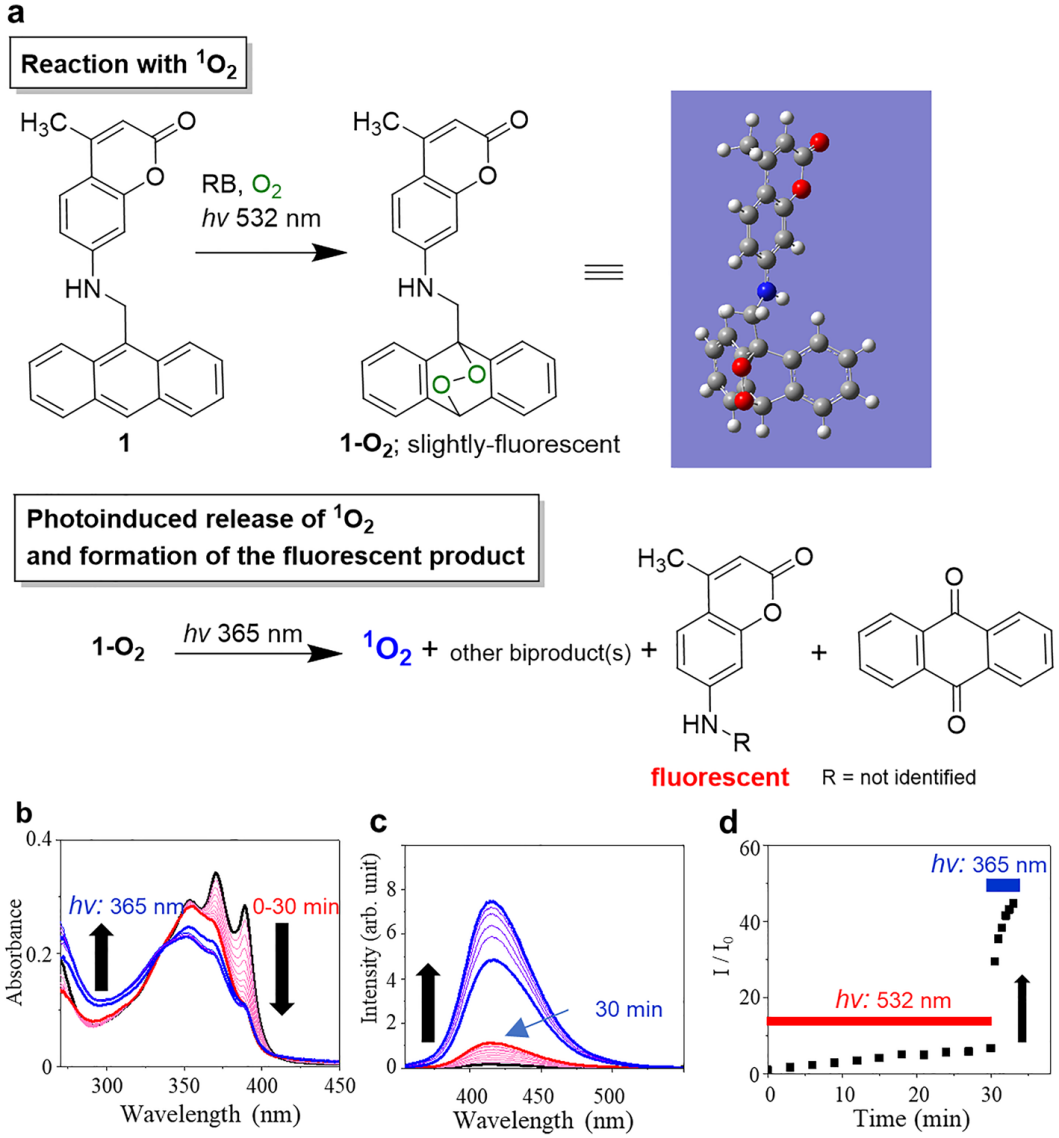
Trapping and photoinduced fluorogenic sensing and release of ^1^O_2_. (**a**) Schemes of the two-step reactions of **1** and ^1^O_2_. The 3D molecular image shows a DFT-optimized structure. (**b**) Absorption spectra of a solution containing **1** (10 µM) and RB (5.0 µM) in DMF before (black line) and after every 5 min of the photosensitization (red line), followed by photoactivation by UV illumination (365 nm, 1.0 mW cm^−2^) (blue line). (**c**) Fluorescence spectra (*λ*_ex_: 340 nm) of **1** in the same conditions as in (**b**). (**d**) Time-trace of the peak emission intensities at 420 nm in (**c**). The red and blue bars indicate the illumination time points by 532 and 365 nm lights, respectively.

The anthracene-based electron donor–acceptor molecule possessing a coumarin chromophore (**1**) (Fig. 1a) was synthesized by a one-step reaction started from 7-amino-4-methylcoumarin and 9-chloromethylanthracene, and characterized by the spectroscopic methods including NMR spectrometry (see “Methods” section). First, we confirmed that **1** is nonfluorescent because of the intramolecular electron transfer from the anthracene moiety to coumarin moiety of **1**^19,20^. **1** reacts with ^1^O_2_ generated by the photosensitization of Rose Bengal (RB) by green light to form a moderately fluorescent endoperoxide **1**-**O**_**2**_ (Fig. 1). The formation of **1**-**O**_**2**_ was confirmed by 1D and 2D NMR measurements on the isolated product of the reaction. The NMR spectra help us to confirm the formation of **1**-**O**_**2**_. The high-field shift of the signals corresponding to the anthracenyl moiety indicated the breaking of the large pi-conjugation due to the endoperoxide formation, without shifting the signals to the coumarin moiety (Figs. S6 and S7). The observed correlation further supports this assignment among the signals in the NOESY spectra (Fig. S7). One of the characteristic correlations is observed between 4.50 ppm and 6.86 ppm from the methylene protons and the proton attached to the nitrogen atom. Also, a correlation between 4.50 ppm and 7.53–7.56 ppm from the methylene protons and the protons on the anthracenyl moiety evidences the justification of the attribution shown in Fig. S6.

The negative values of the calculated relative free energies of formation at the UB3LYP/6–311 +  + G** level on **1-O**_**2**_ (− 0.66 kcal/mol relative to **1** and ^1^O_2_) also guarantee the feasibility of the formation of **1-O**_**2**_ (Fig. S8). Absorption spectroscopic observations provide information on the reaction products. Figure 1b shows the absorption spectra of **1** recorded as a function of time under the photosensitization of RB. The intensities of the anthracene's vibrational bands at 390 nm and 370 nm are decreased by the ^1^O_2_ mediated oxidation of the anthracenyl moiety^19,26^. After the UV illumination, the absorbance around 290 nm was remarkably increased, suggesting the formation of the endoperoxide **1-O**_**2**_^26^.

Remarkably, the fluorescence quantum yield of **1**-**O**_**2**_ was found to be *ϕ* ~ 0.03 and is not as high as commercially available fluorogenic ^1^O_2_ probe molecules which form the endoperoxide in the fluorescent form (*ϕ* > 0.5)^5,7^ although the original dye part of **1**-**O**_**2**_ (i.e., 7-amino-4-methyl coumarin; Coumarin 120) is highly fluorescent (*ϕ* = 0.62)^19^. This result implies the huge fluorescence intensity enhancement possibility during ^1^O_2_ sensing and the existence of a non-radiative relaxation pathway in the photoexcitation of **1**-**O**_**2**_, which is discussed below. Interestingly, a short-time illumination of UV light (365 nm, 1.0 mW cm^−2^) on both the reaction crude between **1** and ^1^O_2_ or the isolated **1-O**_**2**_ induced the remarkable fluorescence intensity enhancement (Fig. 1c and d). A 45-fold increase in the emission intensity from the starting **1** occurred by 3 min UV light illumination. This change inspired us to understand the formation of the products in the reaction of **1** and ^1^O_2_.

The changes in the absorption and emission characteristics between **1-O**_**2**_ and the final product(s) suggest that the UV illumination significantly accelerated the change in the molecular structure. Anthraquinone is one of the resultant products after UV illumination due to an oxidative rearrangement^16,27–29^, supported by the ^1^H-NMR spectrum of the isolated **1-O**_**2**_ after UV illumination (Figs. S6c and S6d). The quantification of the decomposed products is estimated to be 2:1 (R-substituted- coumarins:anthraquinone, mol:mol) based on the crude NMR signals after the UV illumination shown in Fig. S6b where the signals in 4.0–5.5 ppm are expected for H_g_ of R-substituted-coumarins, and 8.22 ppm for the four protons of anthraquinone. It is noteworthy that the photoexcitation on the chromophore part, not on the endoperoxide like in the previous study^16,28^, triggers the generation of anthraquinone. The ^1^H-NMR spectra showed the characteristic peaks from the coumarin moiety (2.3 and 5.97 ppm) (Fig. S6c). However, the signals in the aromatic region showed complexity which indicates the formation of several products derived from the coumarin moiety. We successfully isolated the intermediate of **1-O**_**2**_ under dark conditions, which affords fluorescent product enabling temporally controlled fluorogenic sensing of ^1^O_2_ by **1**.

**Figure 2:**
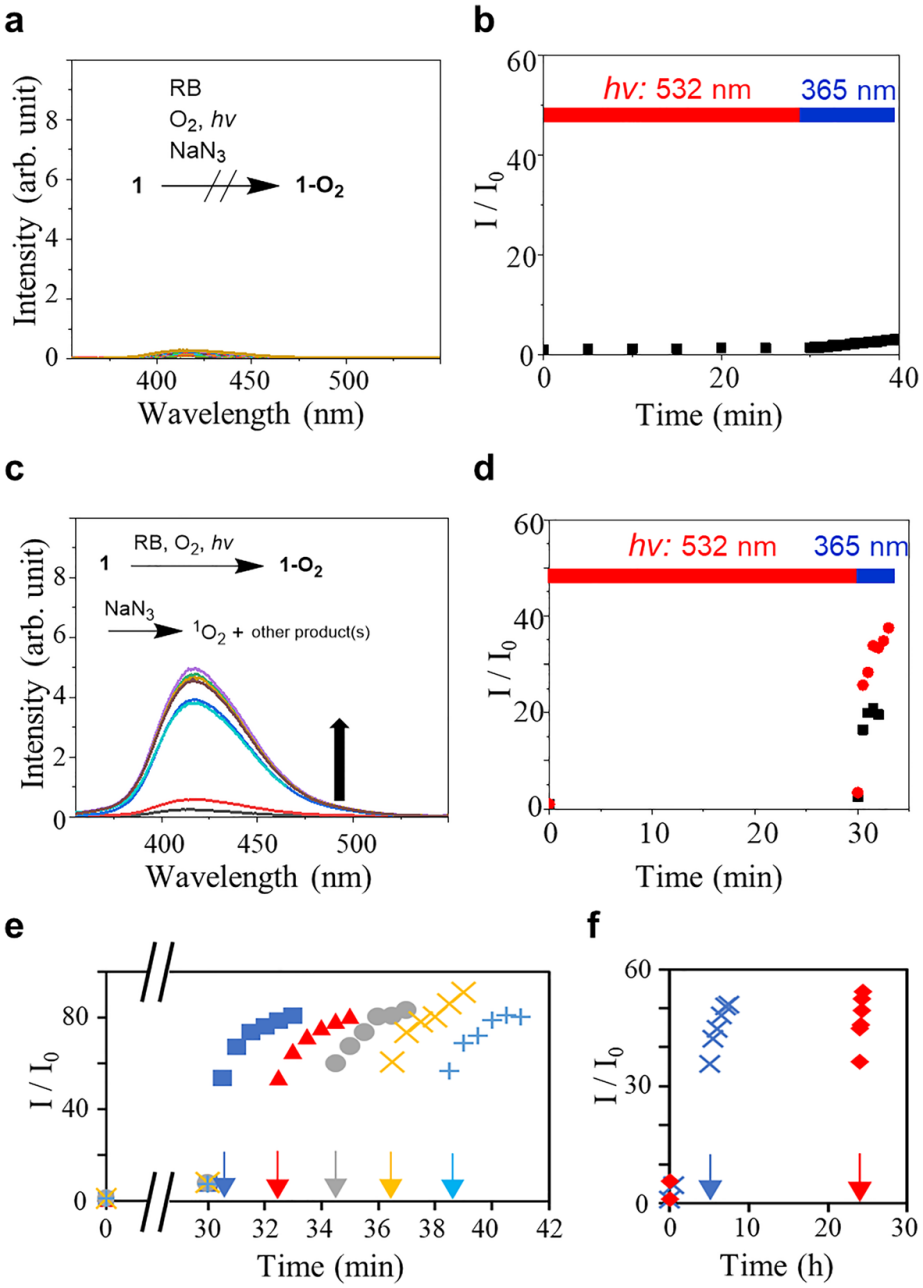
Temporal controlled detection of ^1^O_2_ by **1**. (**a**) Fluorescence spectra (*λ*_ex_: 340 nm) of a solution of 1 (10 µM) and RB (5 µM) before and after every 5 min of photosensitization (532 nm, 50 mW) and photoactivation with the UV light (365 nm, 1 mW cm^−2^) in DMF, and in the presence of ^1^O_2_ scavenger NaN_3_. (**b**) Time-traced relative emission intensities at 420 nm. The red and blue bars indicate the illumination time points by 532 and 365 nm lights, respectively. (**c**) Fluorescence spectra of a solution of **1** and RB in DMF before and after the photosensitization (532 nm, 50 mW) for 30 min, followed by the addition of NaN_3_ (150 µM) and photoactivation with the UV light (365 nm, 1 mW cm^−2^). (**d**) Time-traced relative emission intensities at 420 nm of the sample solutions containing NaN_3_ (black) and without NaN_3_ as a control experiment (red). (**e**,**f**) The UV-induced changes to the emission intensity after storing **1-O**_**2**_ at different times ranging from 30 min to 24 h. The arrows indicate the starting time point of the UV-light excitation for each condition.

To further verify the reaction mechanism in the UV-induced fluorescence enhancement, we recorded the emission spectra of **1** in the presence of a ^1^O_2_ scavenger NaN_3_ (Figs. 2a-d and S5)^30,31^. Figure 2a shows the emission spectra of the sample solution containing **1** and RB with 15 eq. of NaN_3_. The spectra represent the changes during the photosensitization with the 532 nm laser and the UV illumination. The emission intensity remained unvaried during the 532 nm laser irradiation and almost unchanged by the following UV illumination (Fig. 2b). A similar result was obtained in the experiment using the sample solution purged with argon prior to the illumination (Fig. S3). This result verifies the essential participation of ^1^O_2_ to form **1-O**_**2**_.

Meanwhile, **1-O**_**2**_ showed substantial stability towards the ^1^O_2_ scavenger. To verify this, a sample solution containing **1** and RB was irradiated by the 532 nm laser for 30 min, and afterward, 15 eq. of NaN_3_ were added and illuminated with UV light. The UV-induced fluorescence intensity enhancement was observed in this case (Fig. 2c and d). Therefore, ^1^O_2_ plays an important role only in the formation of **1-O**_**2**_ and does not participate in the UV-light triggered emission intensity enhancement. Furthermore, to investigate the contribution of photodimerization of anthracene moiety for the emission intensity enhancement^10,20^, we examined the photoresponse of **1** under the UV illumination in the presence or absence of RB (Fig. S9). Relative to the UV illumination on **1-O**_**2**_, little change was observed in either case, ruling out the significant contribution of the photodimerization. Thus, the UV light-induced intensity enhancement is due to an intramolecular process, which would be the formation of the fluorescent product rather than photodimerization.

Notably, **1-O**_**2**_ is stable in the dark room temperature conditions for more than 24 h. **1-O**_**2**_ was stored in the dark for various periods after the formation of **1-O**_**2**_ by photosensitization of RB and then irradiated by the UV light. **1-O**_**2**_ was found to be stable, which is evident from the UV-induced enhancement of the emission intensities of the sample stored in the dark for 24 h or longer (Fig. 2e and f). We examined its thermal stability by heating it at 100 °C for 30 min under air. The emission spectra remained unchanged after the heating, indicating the high thermal stability of **1-O**_**2**_ (Fig. S10).

### DFT calculations correlated with the fluorescence uncaging of 1-O_2_

**Figure 3:**
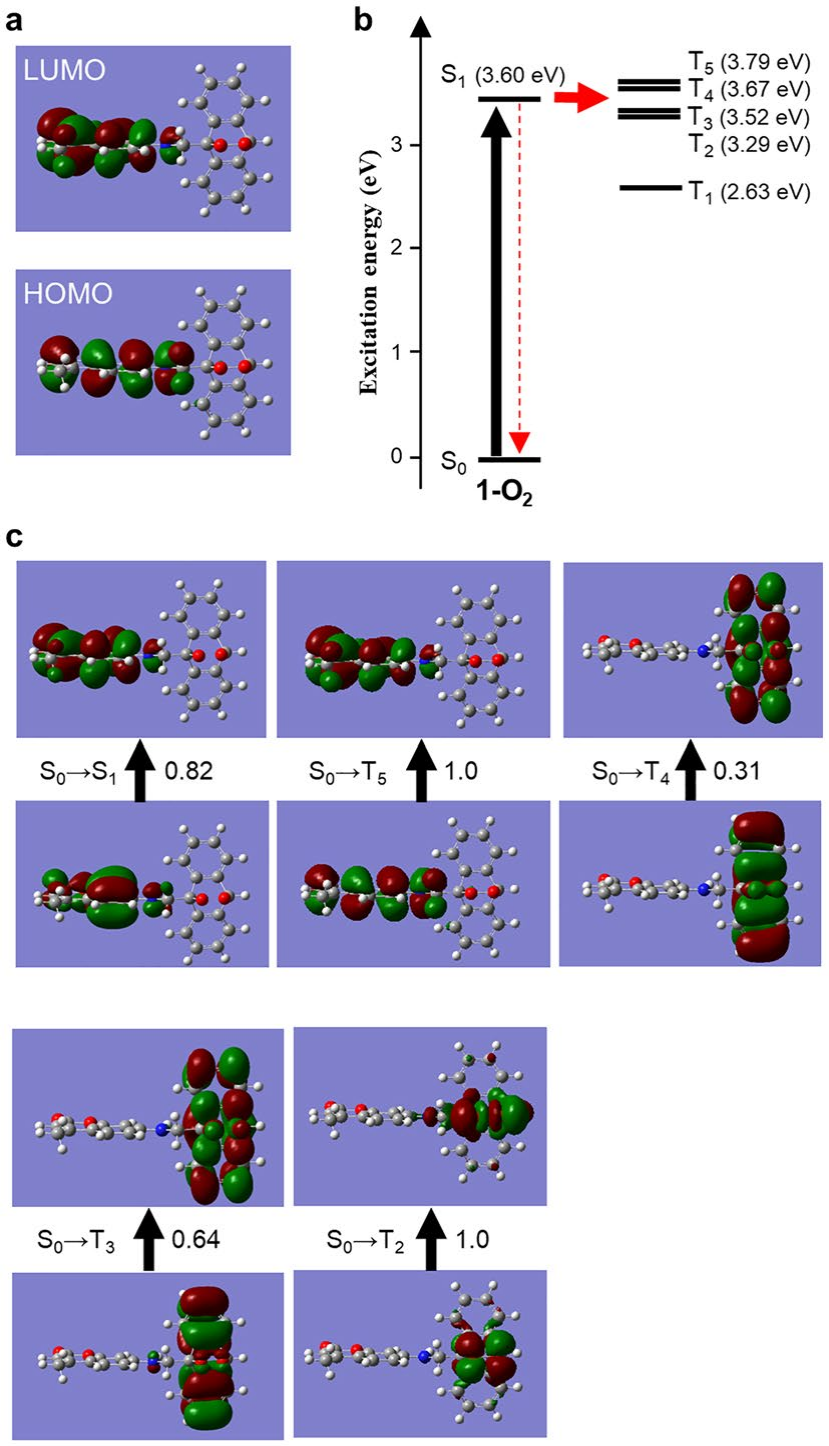
DFT calculations on **1-O**_**2**_ at the UB3LYP/6–311 ++G** level of theory with self-consistent reaction field (SCRF) where DMF is the solvent. (**a**) HOMO and LUMO. (**b**) Energy diagrams of the calculated excited states. (**c**) Natural transition orbitals (NTOs) of the most probable transitions. Black arrows indicate the direction of the transition. The accompanied values indicate the coefficients that correlate with the transition probability, where 1 and 0 are the most and worst probable.

Next, the reasons for the weak fluorescence of **1-O**_**2**_ were studied through the DFT calculations. The localization of HOMO and LUMO on the coumarin moiety of **1-O**_**2**_ supports the most feasible transition in these orbitals through the photoexcitation (Fig. 3a). It is also inductive the intramolecular electron transfer is not feasible because the anthracenyl moiety is not involved in the frontier orbitals^32^. To understand the excited states and the photoinduced transition pathway, the excitation energies and natural transition orbital (NTO) analyses were studied (Fig. 3b and c). NTOs, known to describe compact frontier orbitals^25^, can represent one-electron properties associated with the electronic transition^33^. Figure 3b and c respectively show the energy diagrams of the excitation energy of each excited state and the most plausible electron transition from the highest occupied NTO (HONTO; Fig. 3c, lower, Fig. S10) to the lowest unoccupied NTO (LUNTO; Fig. 3c, upper) in each transition from the ground state (S_0_) of the molecule. The calculations on **1-O**_**2**_ predict the several triplet excited states (T_2_–T_5_), which may possess comparable energy levels to the S_1_ state (Fig. 3b). It is reported that the energy gap of  < 0.37 eV is sufficient to facilitate the intersystem crossing (ISC) via molecular vibrations at room temperature^33^. Furthermore, the contribution of the electrons on the non-bonding nitrogen atom facilitates the ICS according to the El-Sayed rule^34^, which supports favorable transition in ^1^nπ* → ^3^ππ*. Such electron in charge is seen on the nitrogen atom in the both NTO of **1-O**_**2**_ (in the cases of S_0_ → S_1_ and S_0_ → T_5_) and HONTO of **1-O**_**2**_ (S_0_ → T_3_) (Fig. 3c). Consequently, it is rationalized the comparable energy levels among S_1_ and T_2_-T_5_ states and the ^1^nπ* → ^3^ππ* transition originating from the electrons on the nitrogen atom can play a crucial role in the efficient intersystem crossing which leads to the caging of emission in **1-O**_**2**_.

To further elucidate the role of the molecular structure of **1** on the formation of **1-O**_**2**_, we examined different substituents on the 9th position of anthracene (Fig. S1). In 9-methylanthracene, only decreases in the emission intensity and absorbance were detected by the photosensitization and the following UV illumination (Fig. S12). This result supports that the coumarin moiety plays an important role in the enhancement of the emission. To rule out the contribution of the hydrogen atom in the amino group, we examined an *N,N*-dialkylated derivative of **1** (**2**) (Figs. S1 and S2). The UV-induced fluorescence enhancement was observed in **2** as in the case of **1** (Fig. S13). These results suggest that the contribution of the hydrogen atom is negligible. The anthracene framework would play a crucial role in achieving the UV-induced emission intensity enhancement. Furthermore, the DFT calculations conclude that the substituted anthracenyl moiety contributes to the unique low fluorescence of **1-O**_**2**_, which is definitively different from the other reported donor–acceptor type ^1^O_2_ sensor molecules reported so far. Therefore, it is an excellent example showing the fluorescence on/off switching property achieved by the excited-state engineering of molecules.

### The UV-induced uncaging of ^1^O_2_ in 1

**Figure 4:**
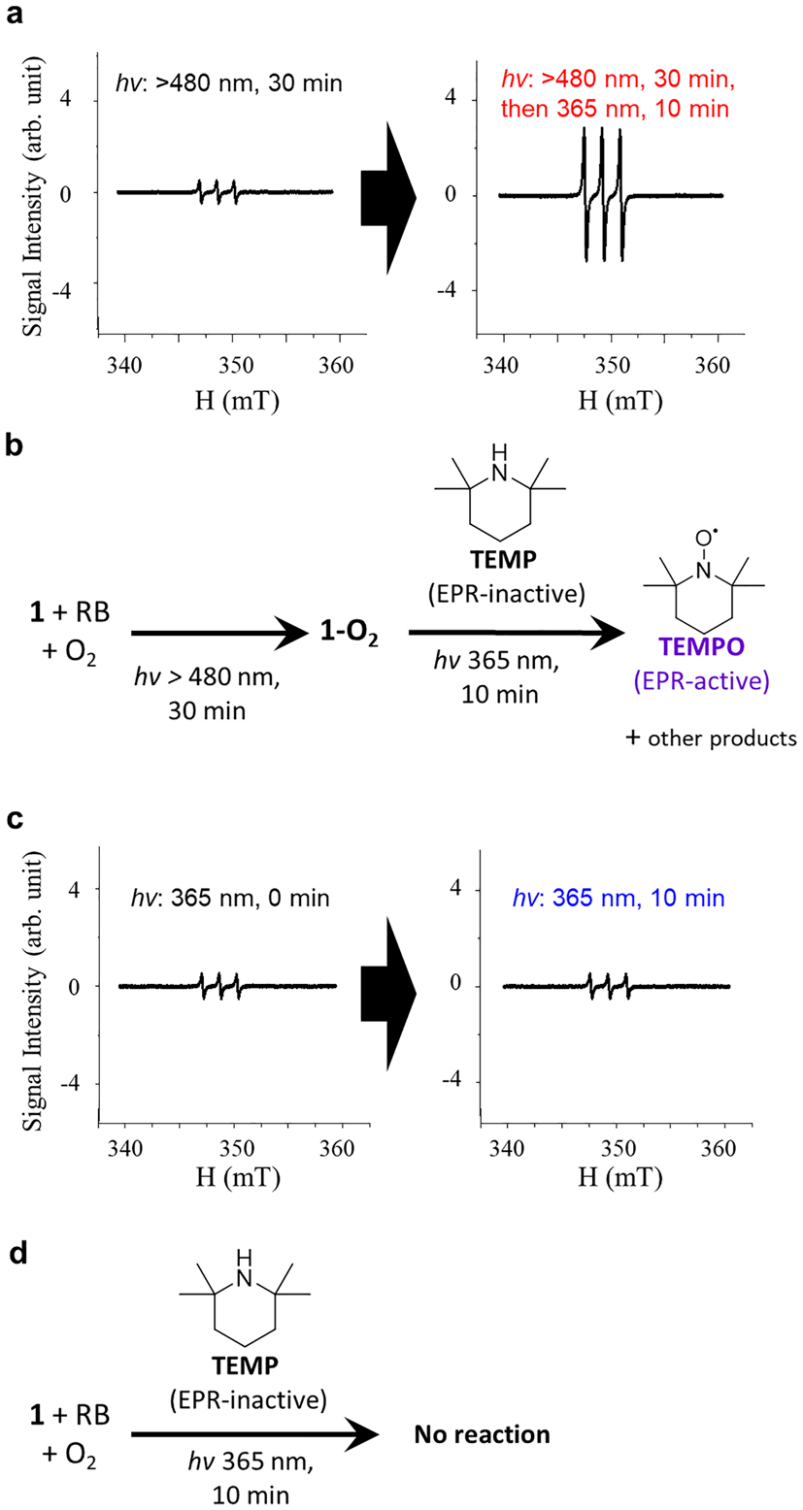
EPR measurements on the sample solution of **1** and RB.** (a**) EPR spectra of the sample solution of **1** and RB (2:1, mol/mol) recorded under the illumination by a xenon lamp (long-pass filter for 480 nm, 30 min, 50 mW), and then TEMP was added. The spectra were recorded: (left) before and (right) after the UV illumination (365 nm, 10 min, 2 mW cm^−2^). The triplet signal seen in the left figure corresponds to the residual signal from TEMPO in the TEMP. (**b**) The corresponding reaction scheme for (**a**). (**c**) EPR spectra of the control experiment only with the UV illumination of a solution of **1**, RB, and TEMP (2:1:1000, mol:mol:mol): (left) before and (right) after the UV illumination (365 nm, 10 min, 2 mW cm^−2^). (**d**) The corresponding reaction scheme for (**c**).

EPR is one of the most widely employed techniques to study the reactions involving ^1^O_2_^35,36^. Remarkably, the UV (365 nm) illumination triggers ^1^O_2_ release in parallel with the fluorescence intensity enhancement, as evidenced by the EPR results (Fig. 4) correlated with the fluorescence and absorption results. A solution containing **1** and RB was illuminated in the presence of a spin probe TEMP, which has the sterically hindered amine to monitor ^1^O_2_ since the oxidation of the probe generates an EPR-active *N*-oxyl radical, TEMPO^36^. The EPR spectra were recorded before and after UV illumination following the photosensitization by RB. A remarkable enhancement of the EPR signal intensity, which indicates the formation of TEMPO, was observed after the UV illumination (Fig. 4a and b). The control experiments without the photosensitization of RB did not give any EPR signal enhancement triggered by the UV illumination (Fig. 4c and d).

We also verify that neither **1** nor RB generates ^1^O_2_ under the UV illumination (Fig. S14), and TEMP itself does not produce or release ^1^O_2_ (Fig. S15). Moreover, the same phenomena, the ^1^O_2_ detection only after the photosensitization of RB in the presence of **1**, were also observed by using SOSG or SiDMA (Figs. S16 and S17). Because the ^1^O_2_ detection by SOSG was also observed with removing O_2_ after the photosensitization, the uncaging of ^1^O_2_ from **1-O**_**2**_ was indicated (Fig. S17b). Thus, it was concluded that the phototriggering releases the caged ^1^O_2_ in 1**-O**_**2**_, along with the formation of fluorescent products (Figs. 1a and S6). Also, we used the commercial sensor SiDMA for examining the ^1^O_2_ release. The absorbency of SiDMA is decreased and disappeared (Fig. S18).

It should be noted that the applied wavelength of light is significantly longer (*hv*: 365 nm), at which anthracene endoperoxide does not possess optical absorption, than those in the previous reports showing the phototriggered release of ^1^O_2_ by the illumination (*hv*: 282 nm)^16^ on the anthracene moiety^16,26^. Furthermore, the release was also detected by two-photon excitation. The linked dye molecule of **1**, coumarin, would change the usable wavelength of light for the phototriggered release from the endoperoxide. The enhancement factor of the EPR signal is ca. 50% relative to the control using TEMP and RB without **1** (Fig. S14), which suggests ca. 50% **1-O**_**2**_ photoreleased ^1^O_2_. The remaining 50% **1-O**_**2**_ would convert to the fluorescent coumarin derivative as mentioned above with the ^1^H-NMR measurements or be retrapped by unreacted **1** in the sample solution. The singlet oxygen photo-releasing quantum yield is estimated to be 1.6% based on the absorbed number of photons (320 nmol) and the detected ^1^O_2_ (10.0 nmol × 50% = 5.00 nmol).

### NIR two-photon induced reaction of 1 with ^1^O_2_

The NIR-active molecular systems promise phototherapy, photo-uncaging-mediated drug delivery, and efficient chemical reactions because of the permeability of cells and tissues to NIR light^37–40^. In this context, we investigated the release of ^1^O_2_ from **1-O**_**2**_ under a pumped-pulsed NIR laser activation since coumarins possess a moderate two-photon absorption cross-Section^41^ First, we verified that one-photon excitation under 404 nm laser excitation activated **1-O**_**2**_ (Fig. S19). Then, we examined the fluorescence and molecular structural features of **1-O**_**2**_ during two-photon excitation with an 800 nm pulsed laser (Coherent Mira 900 with peak power at 7.4 × 10^15^ W). Here, after photosensitization of RB in a sample solution containing **1** and RB with the 532 nm, we applied the NIR light. As a result, **1** showed a threefold enhancement in the fluorescence quantum efficiency after 40 min. This enhancement suggests the release of ^1^O_2_ by the two-photon absorption mediated photoactivation of **1-O**_**2**_ (Fig. S20) where the singlet oxygen releasing efficiency is also expected to be ca. 1.6% relative to the induced paired-photons. The NIR light-triggered and temporally controlled release of ^1^O_2_ provides a promising usage of such dyads for site-specific ^1^O_2_ delivery for various fields such as chemistry, material science, and biology.

## Conclusions

We revealed the unique properties of an anthracene-coumarin donor–acceptor system (**1**) during the reactions with ^1^O_2_. The fluorogenic ^1^O_2_ sensing ability of **1** is unlocked by supplying additional energy by low-intensity UV light or low-energy NIR light after the ^1^O_2_ trapping to form **1-O**_**2**_. The intermediate **1-O**_**2**_ is stable against ^1^O_2_ scavengers and high temperatures in the dark. Unlike the reported anthracene-linked molecules in the endoperoxide form, **1-O**_**2**_ is rather nonfluorescent. We attributed the photo-triggered fluorescence intensity enhancement to the molecular rearrangement and unique intersystem crossing in **1-O**_**2**_. A photo-triggered release of ^1^O_2_ was observed by UV/NIR light illumination on the dye molecule in **1-O**_**2**_ with ~ 50% yield using EPR spectroscopy. DFT calculations support that the unique excited states and the molecular orbitals of **1-O**_**2**_ offer temporal control over ^1^O_2_ capturing, storing, releasing, and sensing. The findings in the present study provide valuable information in the photoexcited state engineering to create novel photofunctional molecular sensors. It will also pave the way for the spatially and temporally controlled utilization of ^1^O_2_ in broad areas such as ^1^O_2_-mediated chemical reactions and photodynamic therapy.

## Supplementary Information

Supplementary Information.

## Data Availability

All data generated or analysed during this study are included in this published article and its Supplementary Information file.
