# Supplementary material for: Caging and photo-triggered uncaging of singlet oxygen by excited state engineering of electron donor–acceptor-linked molecular sensors
Source: Sci Rep. 2022 Jul 5;12:11371. doi: 10.1038/s41598-022-15054-4 (PMC9256616; doi:10.1038/s41598-022-15054-4)
Supplement: Supplementary file 1 — Supplementary Information. [file 41598_2022_15054_MOESM1_ESM.pdf]

Supplementary Information

Caging and Photo-triggered Uncaging of Singlet Oxygen by Excited State Engineering of Electron Donor-Acceptor-Linked Molecular Sensors

Devika Sasikumar,[1,†] Yuta Takano,[1,2,†] Hanjun Zhao,[1] Reiko Kohara,[1] Morihiko Hamada,[3] Yasuhiro Kobori[3] and Vasudevanpillai Biju[a,b]

[1]Graduate School of Environmental Science, Hokkaido University, N10, W5, Sapporo, 060-0810, Japan.

E-mail: biju@es.hokudai.ac.jp

[2]Research Institute for Electronic Science, Hokkaido University, N20, W10, Sapporo, 001-0020, Japan.

[3]Department of Chemistry, Graduate School of Science, Kobe University, 1-1 Rokkodaicho, Nada-Ku, Kobe, 657-8501, Japan.

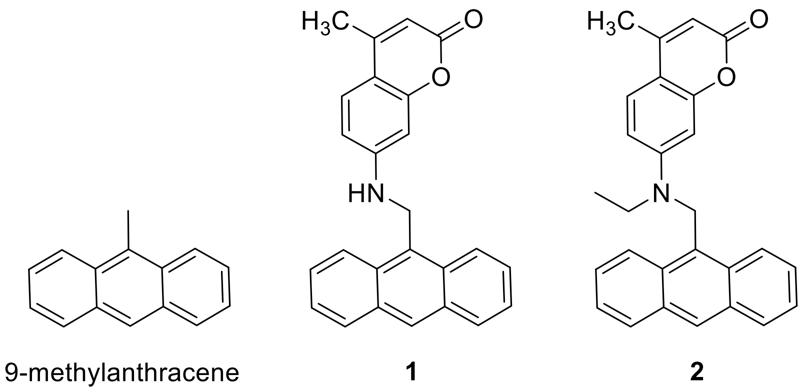

Fig S1. The structures of **1**, **2** and 9-methylanthracene.

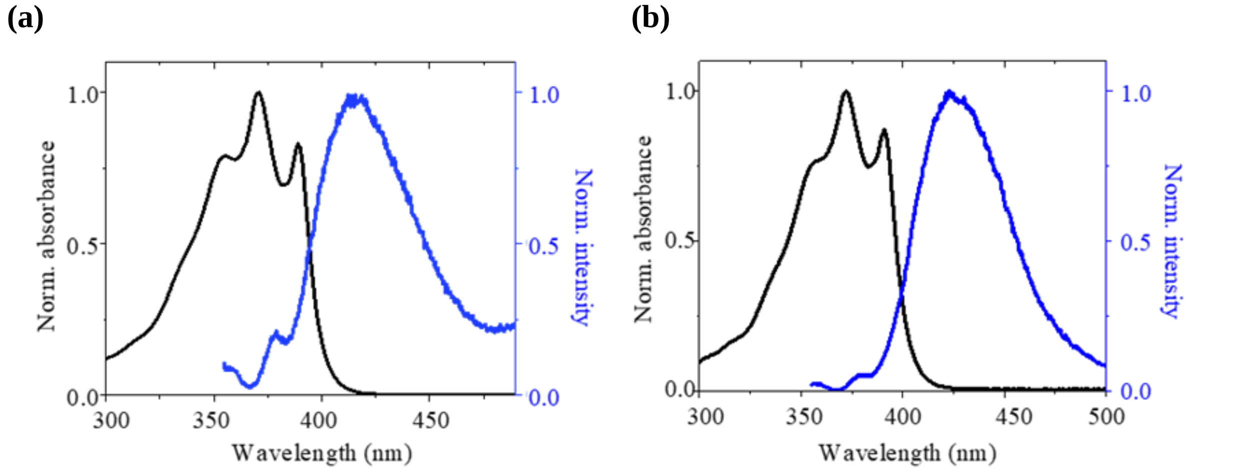

Fig S2. Normalized absorption and FL spectra of a DMF solution of (a) **1** (λ_{ex}: 340 nm), and (b) **2** (λ_{ex}: 340 nm).

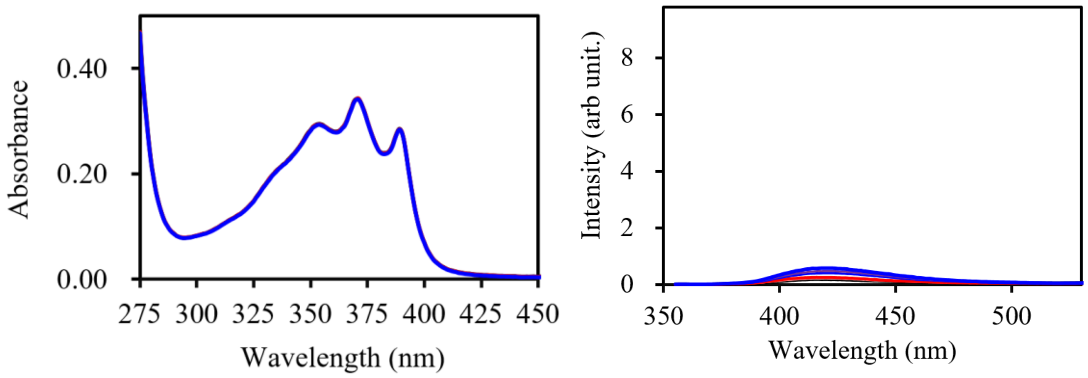

Fig S3. (Left) Absorption spectra and (right) fluorescence spectra (λ_{ex}: 340 nm) of a solution containing **1** (10 µM) and RB (5.0 µM) in argon purged DMF before (black line) and after every 10 min of the photosensitization up to 30 min (red line), followed by photoactivation by UV illumination (365 nm, 1.0 mW cm^{-2}) (blue line) up to additional 5 min. Similar changes to Fig. 2, where a $^1O_2$ scavenger NaN_3 is present, were also observed under these conditions.

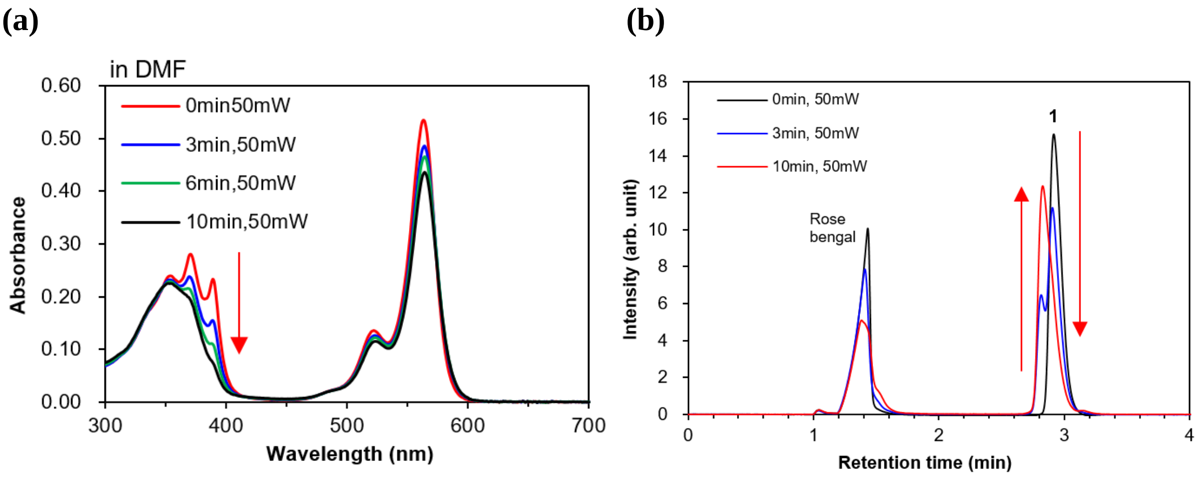

Fig S4. (a) Absorption spectra and (b) HPLC profiles of the reaction mixtures during the 532 nm laser illumination (50 mW) on the mixture of **1** and RB in DMF. Because of the high concentration effect, the reaction completed in a shorter time than those shown in Fig 1. HPLC conditions; Column: C18-MS-II column 4.6 mm I.D. × 250 mm, eluent: DMF 1.0 mL/min, sample loop: 100 µL, inject. vol.: 10 µL, detection λ: 325 nm, temp: 30 °C.

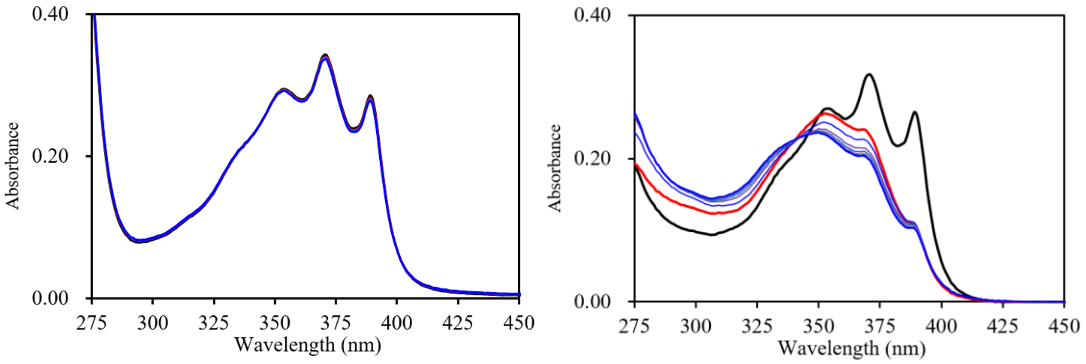

Fig S5. Corresponding absorption spectra in (left) Fig. 2a in which all illuminations were done in the presence of NaN_3, and (right) Fig. 2c in which NaN_3 was added after the photosensitization. Absorption spectra of a solution of **1** (10 μM) and RB (2:1 molar ratio) in DMF before (black line) and after the photosensitization (λ=532 nm, 50 mW) for 30 min (red line), by photoactivation by UV illumination (365 nm, 1.0 mW cm^{-2}) (blue line).

(a)

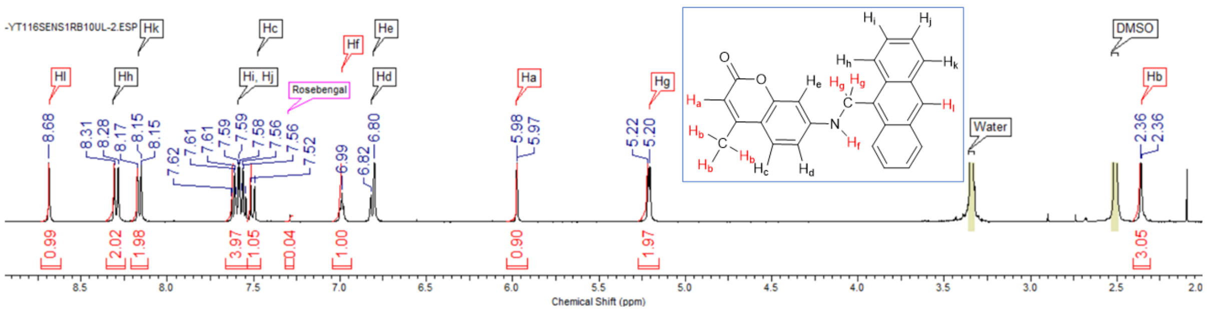

(b)

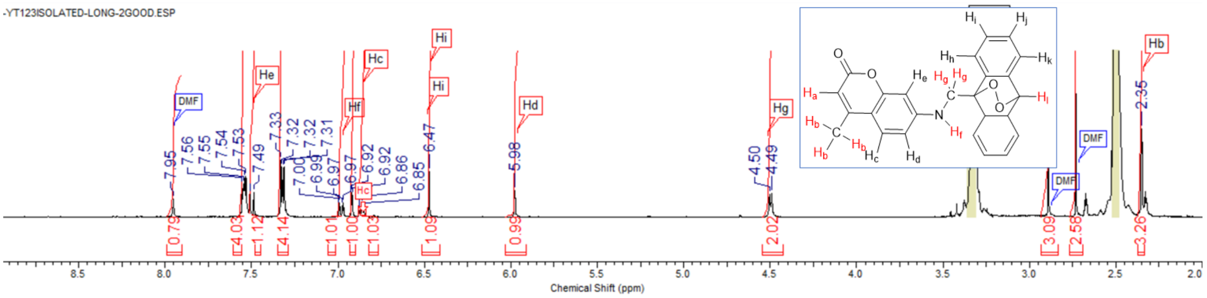

(c)

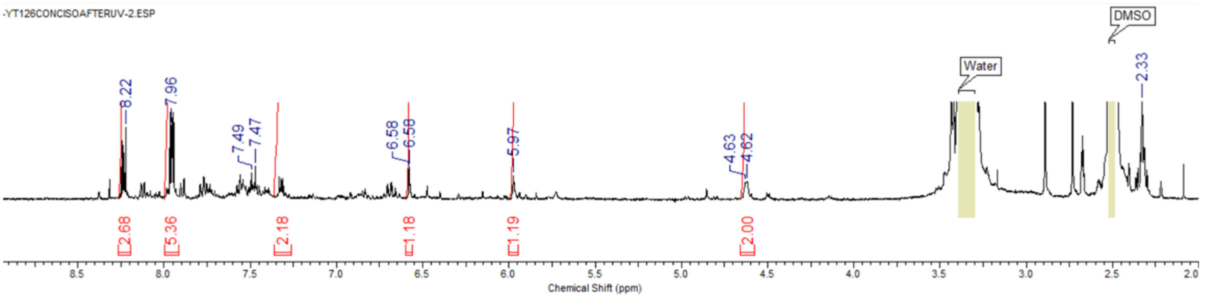

(d)

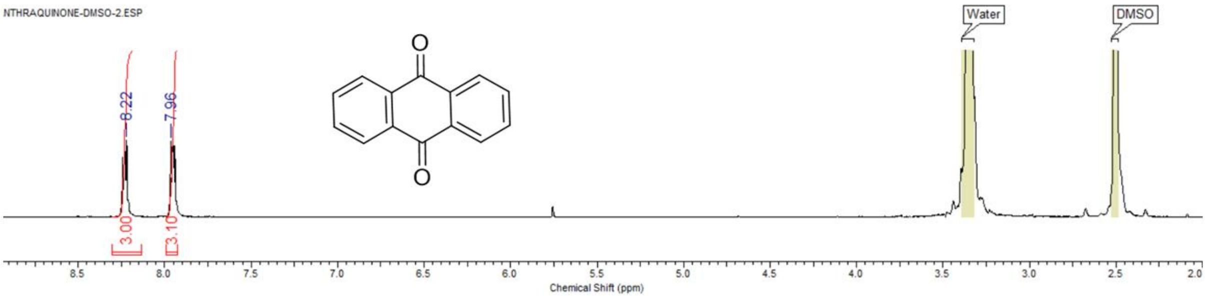

Fig S6. ^{1}H-NMR spectra of (a) **1** and RB, (b) the isolated compound **1-O₂**, (c) the reaction crude after the UV-illumination on **1-O₂**, and (d) anthraquinone, in DMSO-d₆ at 400 MHz. Insets show the structures and corresponding labels of **1** and **1-O₂**.

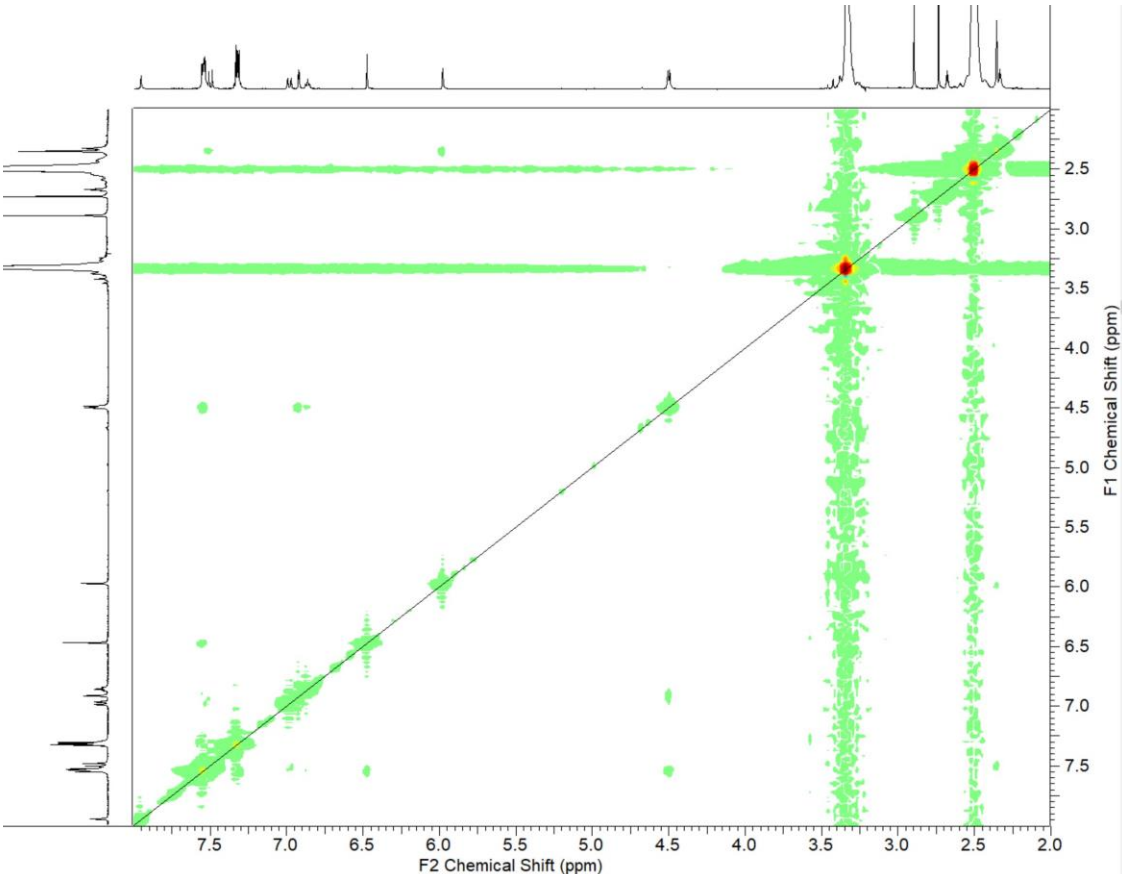

Fig S7. 2D ^{1}H-^{1}H NOESY spectra of the isolated compound (**1-O$_2$**) in DMSO-d$_6$ at 400 MHz.

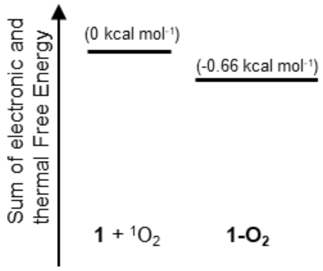

Fig S8. Calculated relative free energies of formation of **1** and **1-O$_2$** at the UB3LYP/6-311++G** level of theory with Self-Consistent Reaction Field (SCRF) where DMF as the solvent.

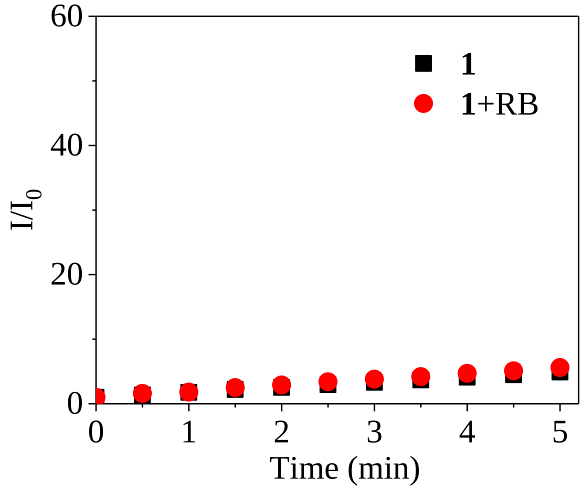

Fig S9. Time-traced relative FL intensities (at λ=420 nm) of DMF solution of **1** (10 μM) before and after irradiation under UV lamp (365 nm, 1 mW cm^{-2}) in the presence (red trace) and absence (black trace) of RB (5 μM) for 5 min.

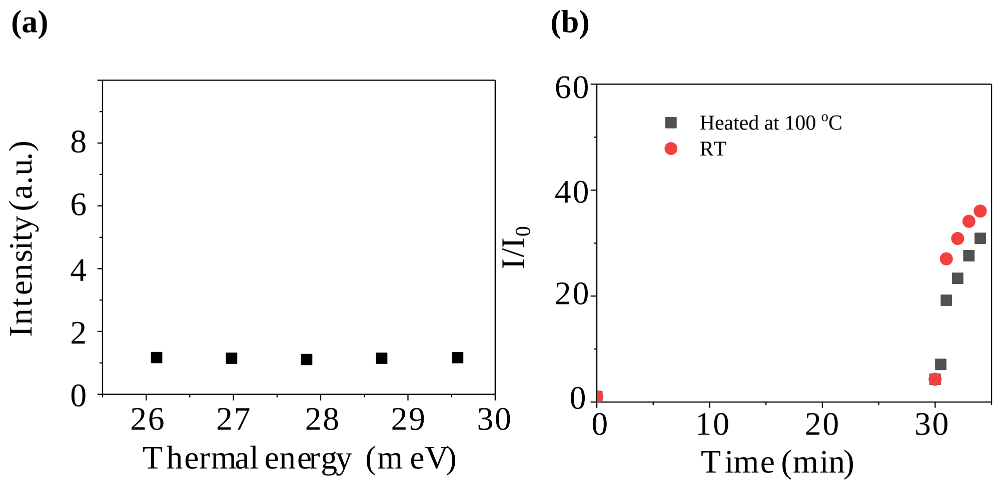

Fig S10. (a) The FL intensity (λ_{ex}: 340 nm) of the solution of **1** (10 μM) and RB (2:1 molar ratio) before and after photosensitization (λ=532 nm, 50 mW) followed by thermal activation, plotted as a function of thermal energy FL spectra. (b) Time-traced relative FL intensities (at λ=420 nm) of a solution of sensor **1** (10 μM) and RB (2:1 molar ratio) in DMF before and after 30 min of photosensitization (λ=532 nm, 50 mW); then the solution was heated to 100 °C and illuminated with UV light (365 nm, 1 mW cm^{-2}). The black traces represent the sample solution's FL response after heating up to 100 °C followed by UV activation. The red trace corresponds to the control experiment conducted at rt.

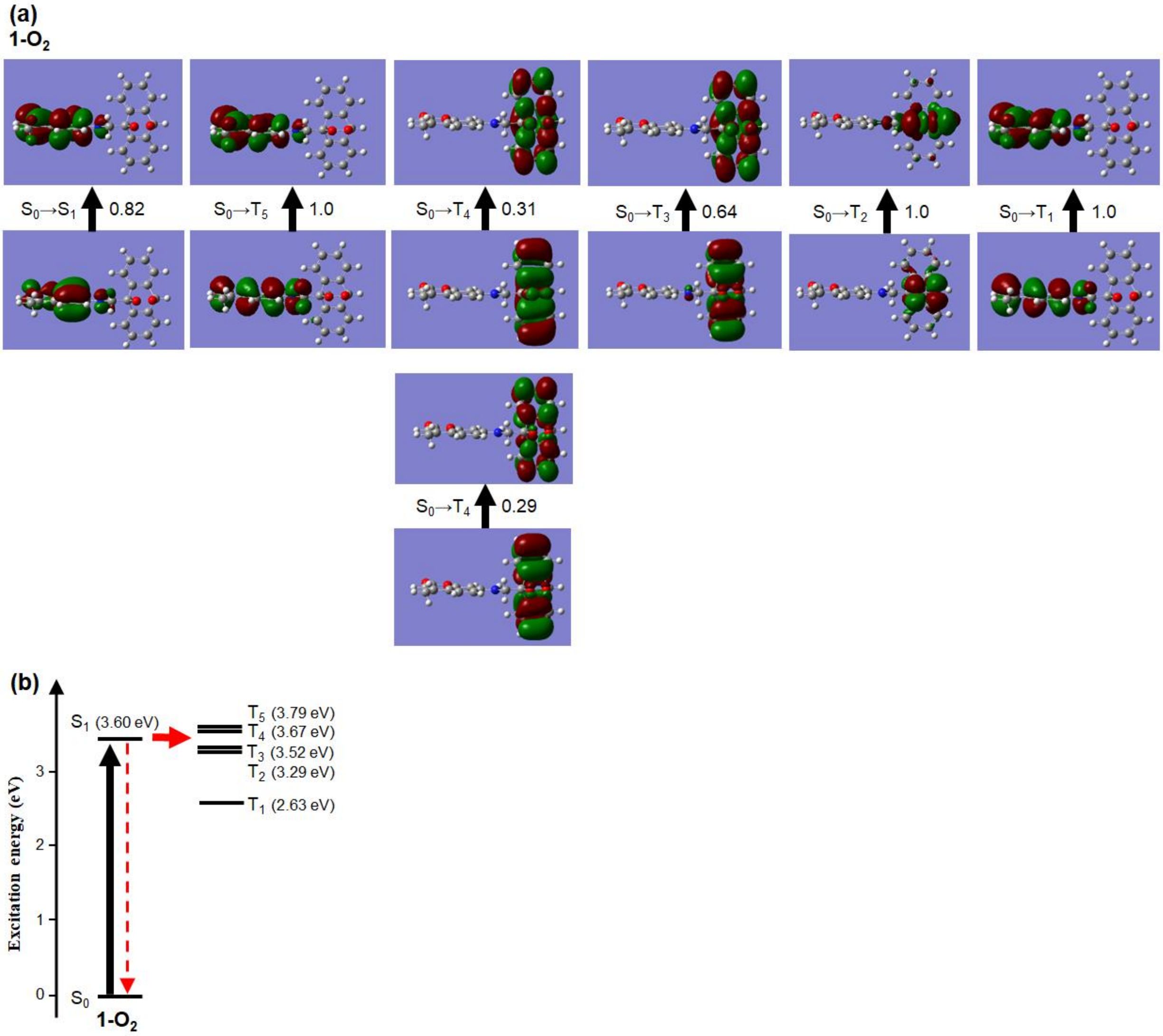

Fig S11. (a) NTOs of the probable transitions of **1-O**$_2$. The second most probable transition is shown for S_0-T_4 of **1-O**$_2$ because the coefficient corresponding to the transition probability is less than 0.5 for the first most probable one. Black arrows indicate the direction of the transition. The accompanied values indicate the coefficients that correlate with the probability of the transition, where 1 and 0 are the most and worst probable. (b) Energy diagrams of the calculated excited states are also shown in Fig. 3 in the main text, at the UB3LYP/6-311++G** level of theory with Self-Consistent Reaction Field (SCRF) where DMF is the solvent.

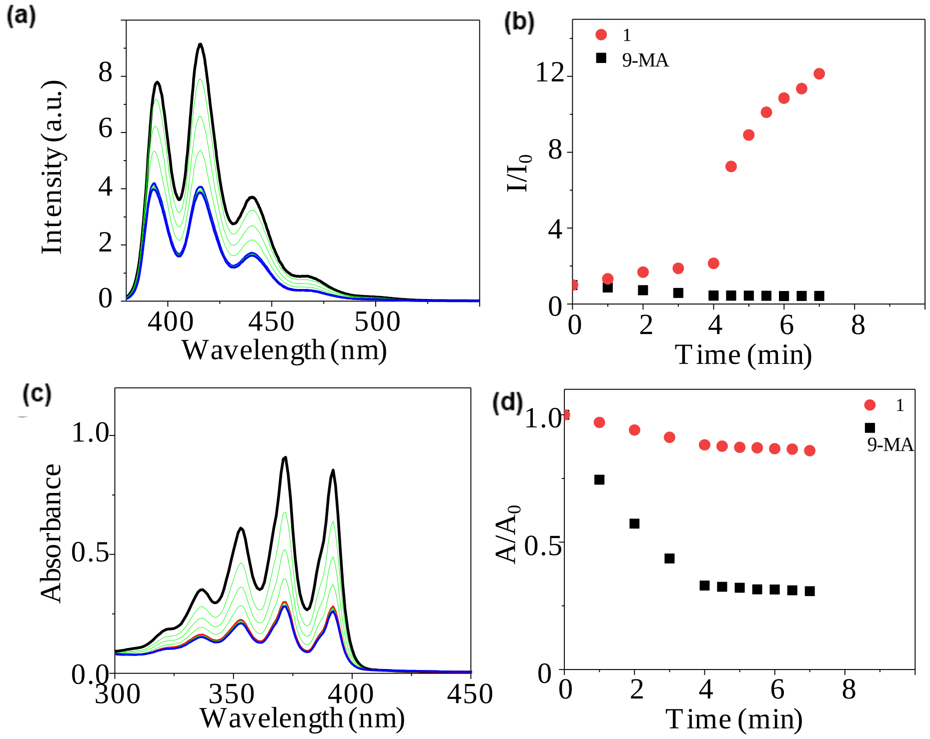

Fig S12. (a) FL (λ_{ex}: 350 nm), and (c) absorbance spectra of a solution of 10 μM of 9-methylanthracene (9-MA) and RB (2:1 molar ratio) in DMF before and after the photosensitization (λ=532 nm, 50 mW) for 4 min, followed by photoactivation with UV (365 nm, 1 mW cm^{-2}) for 3 min. (b) Time-traced relative FL intensities (at λ=420 nm). The black squares represent the FL response of 9-methylanthracene, and the red circles correspond to that of **1**. (d) Time-traced relative absorbance (at λ=370 nm) black trace and red trace represents 9-methylanthracene and **1** respectively.

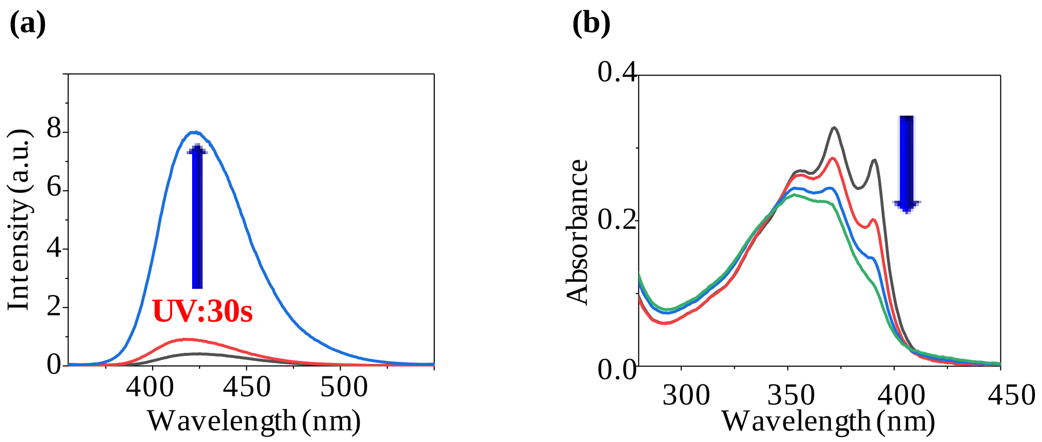

Fig S13. (a) FL (λ_{ex}: 340 nm), and (b) absorption spectra of a solution of sensor **2** (10 μM) and RB (2:1 molar ratio) in DMF before and after the photosensitization (λ=532 nm, 50 mW) for 30 min, followed by photoactivation with UV (365 nm, 1 mW cm^{-2}) for 1 min.

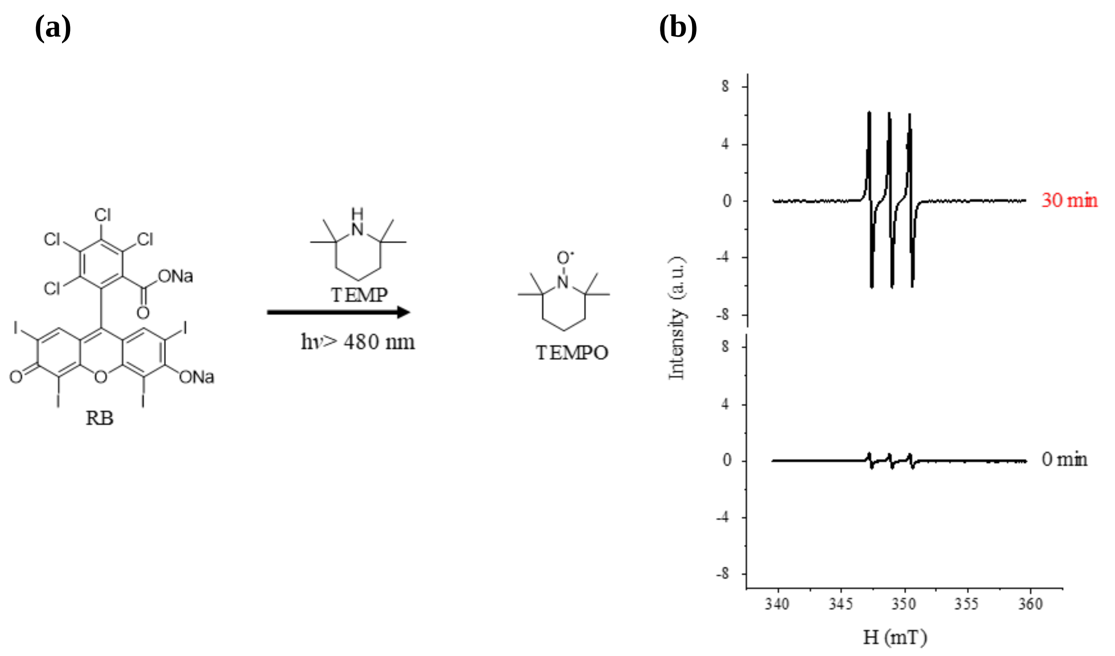

Fig S14. (a) A scheme of the photosensitized generation of $^1O_2$ by RB and oxidation of TEMP to produce TEMPO. (b) The EPR spectra of a solution containing 5 μM RB and 5 mM TEMP in DMF before and after 30 min illumination with a xenon lamp (>480 nm long-pass filter, 50 mW cm-^{2}).

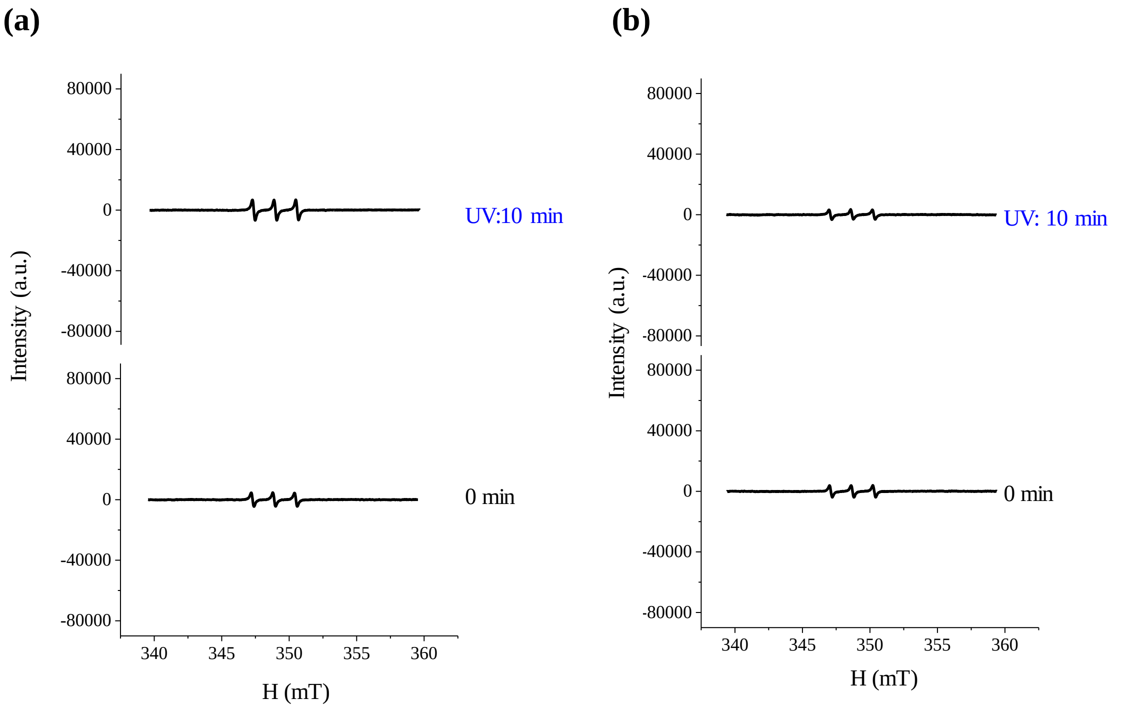

Fig S15. (a) The EPR spectra of a solution containing 5 μM RB and 5 mM TEMP in DMF before and after 10 min illumination with a UV LED (365 nm, 2 mW cm^{-2}). (b) The EPR spectra of a solution containing 10 μM of **1** and 5 mM of TEMP in DMF before and after 10 min illumination with a UV LED (365 nm, 2 mW cm^{-2}).

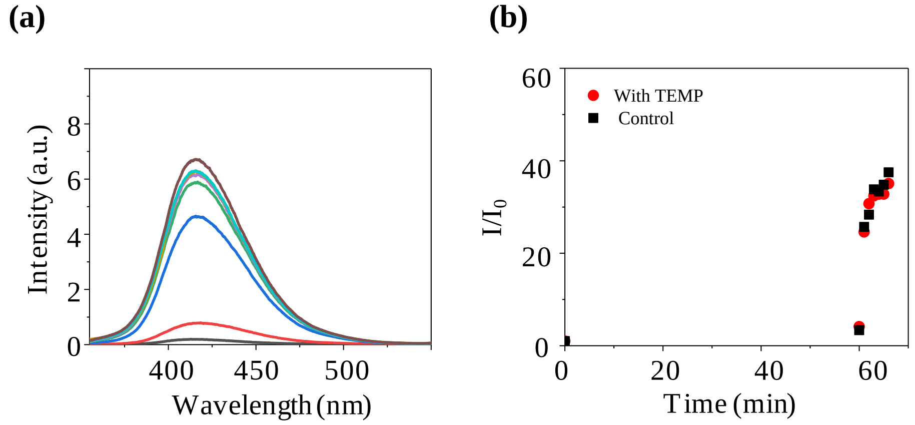

Fig S16. (a) FL spectra (λ_{ex}: 340 nm), of a solution of **1** (10 µM) and RB (2:1 molar ratio) in DMF before and after the photosensitization (λ=532 nm, 50 mW) for 30 min, then the $^1O_2$ probe, TEMP (5 mM) was added and photoactivated with UV (365 nm, 1 mW cm^{-2}). (b) Time-traced relative FL intensities (at λ=420 nm): the red traces represent the FL response of the sample solution containing TEMP and the black traces correspond to the control experiment conducted without TEMP.

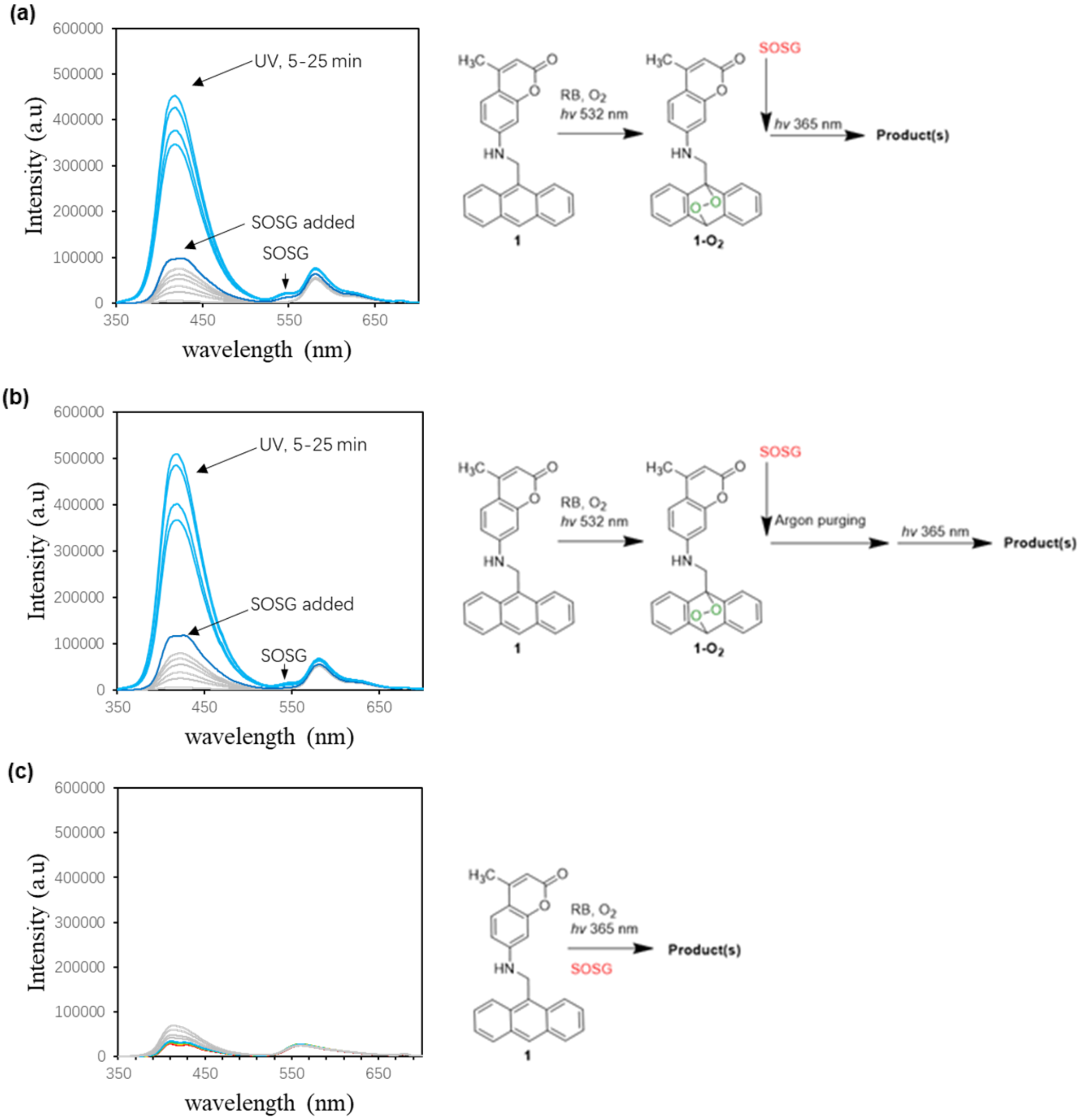

Fig S17. (Left) FL spectra (λ_{ex}: 340 nm), and (right) the corresponding reaction schemes. (a,b) **1** (10 µM) and RB (10 µM) in DMF before and after the photosensitization (λ=532 nm, 50 mW), then the $^1O_2$ probe, SOSG (5.0 µM) was added and photoactivated with UV light (365 nm, 1 mW cm^{-2}) (A) without or (B) with argon purging of the solution. (c) **1** (10 µM) and SOSG (5.0 µM) were added from the beginning and illuminated by the UV light.

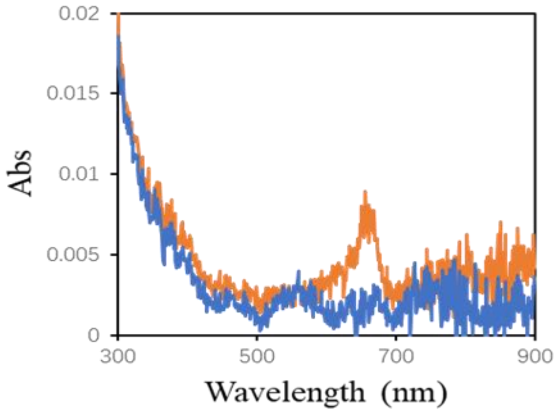

Fig. S18. Absorption spectra of SiDMA before (orange) and after (blue) the UV light illumination (365 nm, 1 mW cm^{-2}) following the photosensitization (λ=532 nm, 50 mW) in the presence of **1** (10 μM) and RB (10 μM). The peak of SiDMA (ca. 660 nm) disappeared after the UV illumination of **1-O$_2$**, showing the reaction of the released singlet oxygen with SiDMA. A low concentration SiDMA (2 μg/mL) sample was used because of the limited commercial availability.

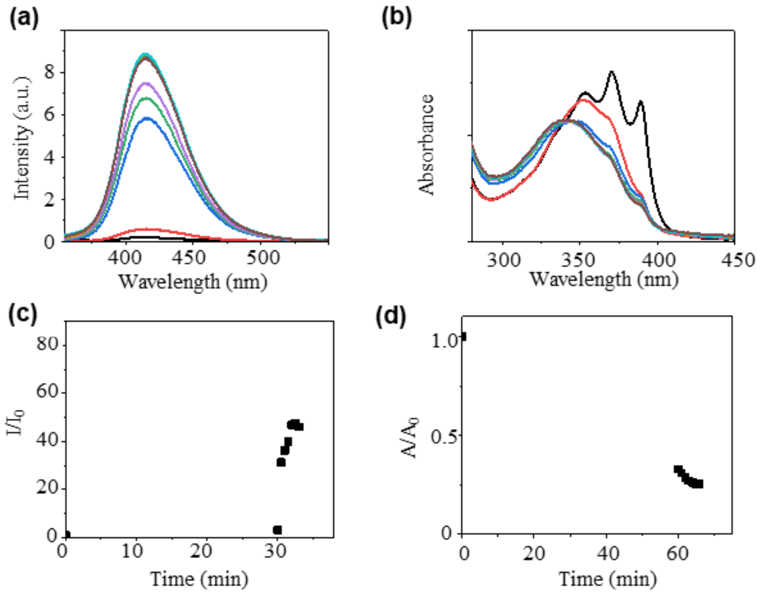

Fig S19. (a) FL (λ_{ex}: 340 nm), and (b) absorption spectra of a solution of **1** (10 μM) and RB (2:1 molar ratio) in DMF before and after the photosensitization (λ=532 nm, 50 mW) for 30 min, followed by photoactivation with 404 nm continuous-wave laser (600 mW) for 3 min. (c) and (d) are Time-traced relative FL intensities (at 420 nm) and relative absorbances (at 370 nm) of **1** to show the enhancement in FL during 404 nm laser excitation, respectively. The black squares correspond to the FL enhancement under 404 nm laser excitation, and blue circles compare FL enhancement under UV light (365 nm, 1 mW cm^{-2}).

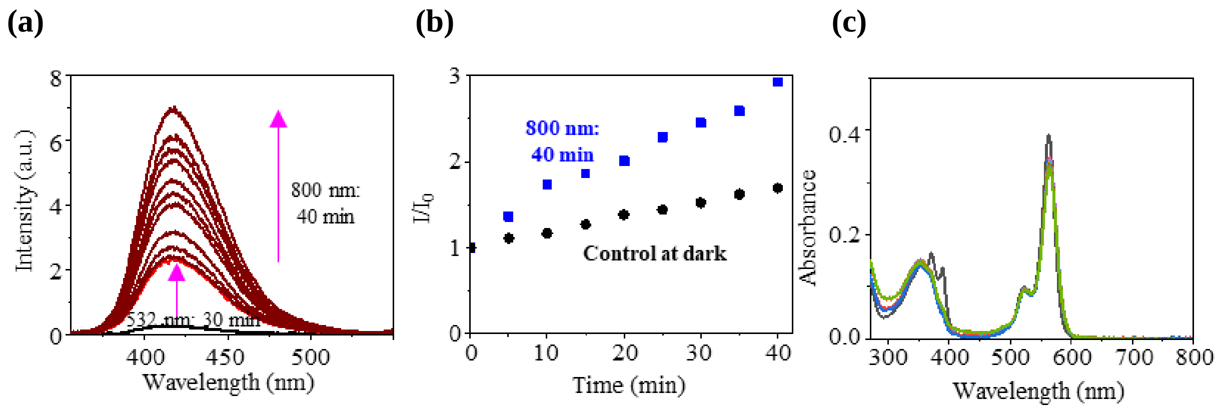

Fig S20. (a) FL (λ_{ex}: 340 nm), and (c) absorption spectra of a solution of **1** and RB (2:1 molar ratio) in DMF before and after photosensitization (λ = 532 nm, 50 mW) and photoactivation with NIR light (800 nm, 540 mW and peak power at 7.42 x 10^{15} W) for 40 min (5 min intervals). (b) Time-traced relative FL intensities of **1** at 420 nm, showing the FL intensity enhancement during NIR activation. The blue squares correspond to the emission intensity enhancement under NIR activation, and the black circles correspond to the temporal changes in FL under dark at room temperature.
